# Spontaneous and instant formation of highly stable protein–nanoparticle supraparticle co-assemblies driven by hydrophobic interaction†

**DOI:** 10.1039/c9na00328b

**Published:** 2019-09-26

**Authors:** Xiaoya Yu, Xiao Liu, Wanchuan Ding, Jun Wang, Gang Ruan

**Affiliations:** Department of Biomedical Engineering, College of Engineering and Applied Sciences, Nanjing University China gangruan@nju.edu.cn; Institute of Materials Engineering, College of Engineering and Applied Sciences, Nanjing University China; Collaborative Innovation Center of Chemistry for Life Sciences, Nanjing University China; Jiangsu Key Laboratory of Artificial Functional Materials, Nanjing University China

## Abstract

Recently, supraparticle protein–nanoparticle co-assemblies (or ‘supraparticle co-assemblies’ for short) have attracted considerable interest due to their fundamental and technological value. However, it remains challenging to form supraparticle co-assemblies with high stability. Here, we show that using hydrophobic interaction, instead of the previously used electrostatic and van der Waals interactions, as the primary driving force can lead to instant formation of exceptionally stable supraparticle co-assemblies with minimal external energy input. Our formation method of supraparticle co-assemblies simply involves mixing globular proteins (*e.g.*, bovine serum albumin) with hydrophobic nanoparticles (*e.g.*, hydrophobic magnetic nanoparticles and hydrophobic quantum dots) without significant energy input (*e.g.*, sonication or stirring). Upon mixing of hydrophobic nanoparticles and proteins, the formation of supraparticle co-assemblies only takes <1 minute. Further incubation of the mixture for several hours results in a gradual increase of the size uniformity of supraparticle co-assemblies. The formed supraparticle co-assemblies have been colloidally stable for 6 months and counting, and can withstand harsh environments such as basic and acidic pH, high temperature, high dilution, and serum. Co-encapsulation of different sizes/types of nanoparticles is found to be feasible and the co-encapsulation number ratio of different nanoparticles is well-controlled by the feeding ratio. Proof-of-concept studies show the potential of the supraparticle co-assemblies for biological imaging, delivery, and modulation. The combination of very rapid formation, minimal energy consumption, highly stable products, and inexpensive raw materials of this hydrophobic interaction-driven process meets many of the main goals of ‘ideal’ nano-manufacturing. Thus, this process could serve as the foundation of ideal manufacturing of supraparticle co-assemblies.

## Abstract 2

A hydrophobic interaction-driven process is discovered to form highly stable protein–nanoparticle supraparticle co-assemblies.

## Graphical abstract

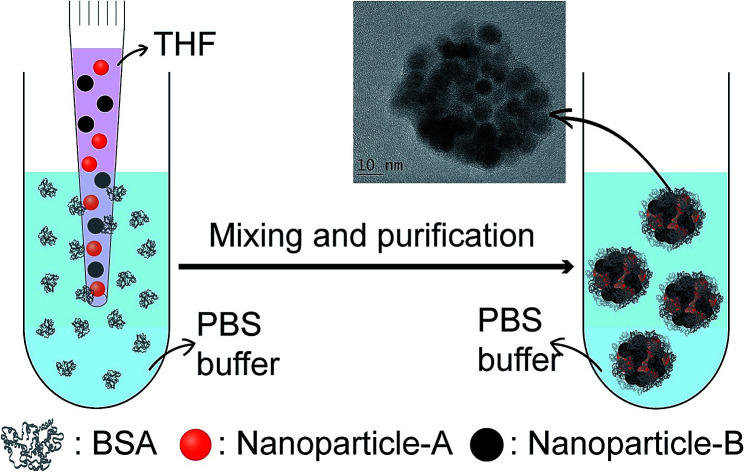

## Introduction

Self-assembly is a cornerstone of the biological world, in which diverse and complex structures are assembled spontaneously from molecular building blocks *via* intermolecular forces.^1^ Great strides have been made to understand self-assembly processes and to design microscopic machinery mimicking biological self-assembly.^2–4^ However, substantial challenges remain in both the fundamental understanding and well-controlled production of self-assembly structures.^2–5^ In particular, assembly of protein molecules has been intensively studied, and some protein assembly structures have been designed and produced.^6^ Inorganic nanoparticles, *e.g.*, semiconductor quantum dots (QDs) and metal nanoparticles, have been used as mimics of globular proteins to spontaneously assemble diverse structures.^7–10^ More recently, hybrid co-assemblies composed of both inorganic nanoparticles and protein molecules as the building blocks have attracted intense interest due to their fundamental and technological value.^11,12^ Terminal assemblies are those with size limitations in all directions.^13–21^ A number of reports have been published recently on terminal supraparticle protein–nanoparticle co-assemblies (or ‘supraparticle co-assemblies’ for short).^13–21^ However, these assemblies often show a lack of colloidal stability or/and non-uniform geometry, thus causing difficulties if they are to be used as nanomaterials.^13–21^

The discovery of the formation of supraparticle co-assemblies between proteins and inorganic nanoparticles was first reported as a surprising observation in 2009 by the Halas group.^13^ After adding negatively charged gold nanoparticles to a solution of lysozyme (positively charged at physiological pH), these authors observed formation of two groups of assembly structures, one being protein–nanoparticle co-assemblies and the other being large protein aggregates.^13^ The observed protein–nanoparticle co-assemblies were highly non-uniform in size and shape.^13^ After this groundbreaking report many studies followed.^14–21^ Most of these studies were fundamental research of the assembly process and structure, while some made efforts to develop protein–nanoparticle supraparticle co-assemblies to be used as a new class of nanomaterials.^14–21^ The Chattopadhyay group studied the use of supraparticle co-assemblies of gold nanoparticles and proteins as drug delivery carriers.^21^ In order to obtain stable supraparticle structures, these authors added an extra layer of proteins after the initial formation of co-assemblies.^21^ In parallel with the research of protein–nanoparticle supraparticle co-assemblies was that of the protein corona of nanoparticles.^22–28^ Although it is well-known that materials can adsorb proteins, it was not until the Dawson group's groundbreaking paper in 2007 that the studies on nanoparticle–protein interactions (forming a protein corona on the nanoparticle surface) really took off.^22^ Since then numerous papers have been published on the protein corona of nanoparticles.^23–28^ In these studies, the nanoparticles with the formed protein corona were usually individual nanoparticles rather than supraparticles (multiple nanoparticles grouped into one particle).^22–28^ In general, it is difficult to use the above bio-nano hybrid structures (supraparticles or individual nanoparticles) as materials, because they are often unstable or/and non-uniform.

We seek to develop protein–nanoparticle supraparticle co-assemblies into a class of nanomaterials and to develop ‘ideal’ nano-manufacturing processes for this class of materials. We note that, in previous reports, the spontaneous formation of supraparticle co-assemblies is largely driven by electrostatic and van der Waals interactions,^13–21^ while some attempts were made to use metal ion-mediated coordinate covalent bonding between proteins and nanoparticles.^29,30^ Hydrophobic interaction has rarely been used previously as the primary driving force for forming supraparticle co-assemblies. Here, we hypothesize that using hydrophobic interaction might yield highly stable supraparticle co-assemblies, considering the fact that globular proteins usually use hydrophobic interaction as the dominant force to form their three dimensional structures.^31^ Thus, in our experiments, we simply mixed model hydrophobic nanoparticles (dissolved in tetrahydrofuran, or THF, a water-miscible organic solvent) and model globular proteins (dissolved in water) without using any significant external energy input such as stirring or sonication. After observing the formation of supraparticle co-assemblies, we performed extensive characterization of the formed assemblies and conducted proof-of-concept studies on their potential to be used as multifunctional nanomaterials.^32–36^

## Experimental methods

### Materials

Hydrophobic quantum dots (QDs) were purchased from Suzhou Xingshuo Nanotech Co., Ltd. Green fluorescence-emitting QDs had a fluorescence peak wavelength at 520 nm, and red fluorescence-emitting QDs had a fluorescence peak wavelength at 620 nm. The surface ligand was trioctylphosphine oxide (TOPO) for both green and red QDs. Bovine serum albumin (BSA), bovine α-lactalbumin (BLA), benzyl ether and oleic acid were purchased from Sigma-Aldrich. Iron(iii) acetylacetonate was purchased from Tokyo Chemical Industry Co., Ltd. 1,2-Hexadecanediol was purchased from J&K Chemical Ltd. (Shanghai). Oleylamine was purchased from Kuer Bio-Engineering Co., Ltd. *N*-Hydroxysulfosuccinimide (NHS) sodium salt, *N*-(3-dimethylaminopropyl)-*N*-ethylcarbodiimide (EDC), 2-(*N*-morpholino)ethanesulfonic acid (MES) buffer and doxorubicin hydrochloride (DOX) were purchased from Aladdin. 3-(4,5-Dimethylthiazol-2-yl)-2,5-diphenyltetrazolium bromide (MTT) was purchased from KeyGENBioTECH. Tetrahydrofuran (THF), hexane and absolute ethanol were purchased from Sinopharm Chemical Reagent Co., Ltd. The RGD peptide (sequence Arg–Gly–Asp) was purchased from ChinaPeptides. Cell lines (HeLa and U87MG) and their culture media were purchased from KeyGENBioTECH.

### Formation of supraparticle co-assemblies

Hydrophobic SPIONs were synthesized based on a well-established method from the literature.^37^ Their surface ligand was oleylamine. Hydrophobic QDs were obtained from a commercial source as described in the Materials section. The surface ligand was trioctylphosphine oxide (TOPO) for both green and red QDs. In a typical formation process of supraparticle co-assemblies, a dispersion of hydrophobic nanoparticles (dissolved in THF, 1 mg mL^−1^, 100 to 800 μL) was injected into a BSA solution (in phosphate buffered saline, or PBS, 2 mg mL^−1^, 3 mL) using a micropipette or a syringe pump. To separate formed supraparticle co-assemblies from free BSA molecules, centrifugation (13 300 rcf, 4 °C, 45 min) was used. After centrifugation, the supernatant was removed and the supraparticle co-assemblies at the bottom were re-dispersed in water or PBS.

### Physico-chemical characterization of supraparticle co-assemblies

The morphology of supraparticle co-assemblies was visualized by transmission electron microscopy (TEM, JEM-200CX, JEOL) without the use of any staining. The dynamic light scattering (DLS) diameter (hydrodynamic diameter) and surface charge (zeta potential) of supraparticle co-assemblies were measured using a Malvern Zetasizer Nano ZS360 instrument. Fluorescence spectra were obtained using a spectrofluorometer (HITACHI F-4600). Thermal gravimetric analysis (TGA) thermograms were recorded using a thermogravimetric analyzer (STA PT 1000, Linseis Instruments) at a heating rate of 10 °C min^−1^ in the temperature range of 30–800 °C under a nitrogen atmosphere.

### Analysis of the number ratio of green QDs to red QDs in a supraparticle assembly with both green and red QDs co-encapsulated

A dispersion of supraparticle co-assemblies (10 μL) was placed between two coverslips and observed with a spinning-disk confocal microscopy system, which consists of an epi-fluorescence microscope (IX-83, 60× oil immersion objective, Olympus, with a halogen lamp as the light source and an excitation wavelength of 488 nm), a spinning-disk (Andor), and an electron multiplying charge-coupled device (EMCCD) camera (Evolve 512, Photometrics). Images were captured using different emission filters to allow the two different fluorescent colors (green and red) to be distinguished. The fluorescence intensity of a fluorescent color of a supraparticle co-assembly was obtained as follows: using the corresponding fluorescence emission filter, a series of 100 images (camera exposure time 400 ms) was continuously captured, and the average fluorescence intensity of each image was calculated using MetaMorph software. The fluorescence intensity of a single QD was obtained by performing the above microscopy-based fluorescence intensity analysis on a dispersion of hydrophobic QDs dissolved in chloroform (10 μL) under the same microscopy conditions (camera exposure time 400 ms, fluorescence intensity of a fluorescent particle averaged over 100 continuous images). The number of QDs of a specific fluorescent color in a supraparticle co-assembly was calculated using the following equation:
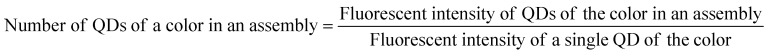


The number ratio of QDs of different colors in a supraparticle co-assembly was then determined using the thus-obtained number of QDs of different colors in an assembly.

### Live cell imaging studies

Cells (HeLa cells or U87MG cells) were seeded at ∼20% confluency in a glass bottom cell culture dish (0.17 mm thickness for the glass bottom) (Nest, China). The cells were cultured in cell culture medium (Dulbecco's modified Eagle's medium + 10% fetal bovine serum) for 12 h in 5% CO_2_ at 37 °C. A dispersion of supraparticle co-assemblies was added. After incubation for 12 h, the medium was removed and the cells were washed with PBS twice. The cells were then imaged with a spinning-disk confocal microscopy system, which consists of an epi-fluorescence microscope (IX-83, Olympus, with a halogen lamp as the light source), a spinning-disk (Andor), and an electron multiplying charge-coupled device (EMCCD) camera (Evolve 512, Photometrics). Image processing and analysis was conducted using MetaMorph and Image J software.

### Cell viability study (MTT assay)

To evaluate the cytotoxicity of a specific formulation, cells were seeded in 96-well plates (Corning Costar, China) with a density of 6000 cells per well. After 24 h of incubation, the cell culture medium was replaced with the formulation for testing (200 μL per well, in complete Dulbecco's modified Eagle's medium). After culturing the cells for 24 h, 20 μL 3-(4,5-dimethylthiazol-2-yl)-2,5-diphenyltetrazolium bromide (MTT) solution (5 mg mL^−1^) and 180 μL DMEM were added to each well and incubated for 4 h at 37 °C. After the medium was removed, the insoluble formazan crystals were dissolved in 150 μL per well of dimethyl sulfoxide (DMSO) and measured spectrophotometrically in an ELISA reader (RT-6000, Rayto, China) at a wavelength of 570 nm. The relative cell viability (%) compared to that of the control well containing the cell culture medium only (in addition to the cells) was calculated from the optical density of the test well divided by that of the control well. All samples were run in quintuplicate.

## Results and discussion

**Fig. 1:**
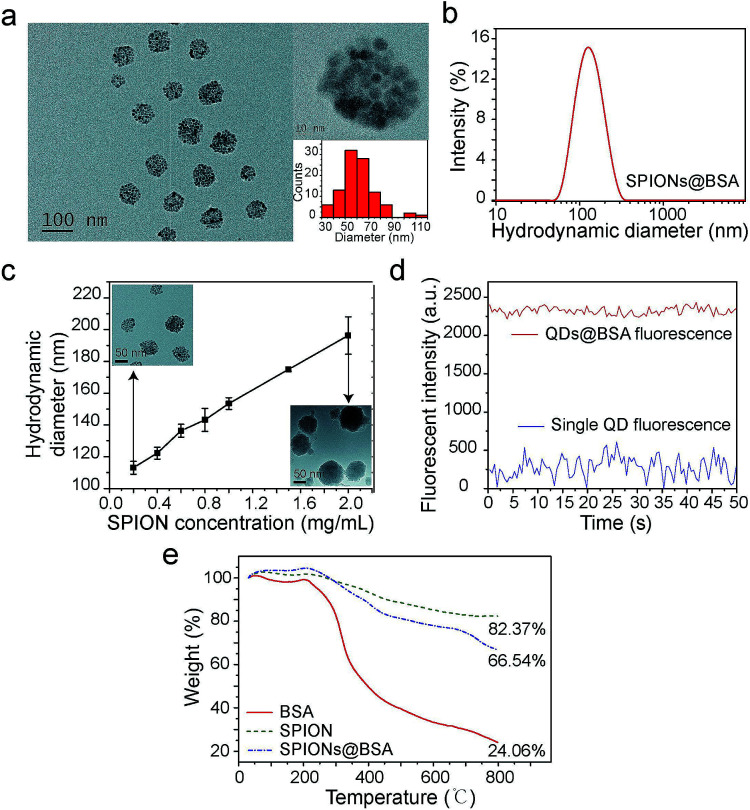
Characterization of supraparticle co-assemblies. (a) TEM image of SPIONs@BSA. (b) DLS results of SPIONs@BSA. (c) Control of the particle size of supraparticle co-assemblies by varying the nanoparticle concentration. The TEM images of the assembly samples with the smallest and the largest DLS sizes are also shown as insets. (d) Enhanced fluorescence intensity of a QDs@BSA supraparticle assembly compared with that of a single QD. (e) TGA results of SPIONs@BSA.

Supraparticle co-assemblies were formed by simply mixing hydrophobic nanoparticles (dissolved in THF, a water-miscible organic solvent) and proteins (dissolved in PBS), without any significant external energy input such as stirring and sonication. The mixing was performed by injecting the hydrophobic nanoparticle dispersion into the protein solution with a micropipette. The hydrophobic nanoparticles examined in the present work include superparamagnetic iron oxide nanoparticles (SPIONs) and semiconductor quantum dots (QDs). The proteins used in the present work primarily include bovine serum albumin (BSA). Transmission electron microscopy (TEM) images confirmed the formation of supraparticle co-assemblies (Fig. 1a). The assemblies were found to be near-spherical in shape (Fig. 1a). The sample image shown in Fig. 1a is that of supraparticle co-assemblies of SPIONs and BSA, which are called SPIONs@BSA here. These assemblies were ∼60 nm in diameter as shown by TEM (Fig. 1a), and ∼115 nm in hydrodynamic diameter as shown by dynamic light scattering (DLS) (three replicate samples, polydispersity 0.2, Fig. 1b). QDs and BSA could also form supraparticle co-assemblies (called QDs@BSA here) with similar sizes (Fig. S1†). Control experiments were conducted to measure the hydrodynamic diameters of BSA (in the absence of the hydrophobic nanoparticles) and hydrophobic nanoparticles (in the absence of BSA). In these control experiments the solvent used was water mixed with a small amount of THF to mimic the solvent conditions in the assembly formation experiments. It was found that BSA alone was ∼9 nm in hydrodynamic diameter, while the hydrophobic nanoparticles in the aqueous environment instantly showed >400 nm hydrodynamic diameter (Fig. S2 and S3,† respectively). The particle size of supraparticle co-assemblies could be varied by changing the concentration of the nanoparticles added (Fig. 1c). A higher nanoparticle concentration was found to yield a larger hydrodynamic diameter (Fig. 1c). In addition, we also examined the effect of operating parameters for injecting the nanoparticle dispersion into the protein solution by using a syringe pump connected to a needle. It was found that the inner diameter of the injection needle and the flow rate of injection affected the assembly size to some extent, yet with an unclear trend (Fig. S4†).

The surface charge of the supraparticle co-assemblies was found to be negative, with the measured zeta potential values being in the range of −30 mV to −14 mV (Fig. S5†). For comparison, the zeta potentials of BSA (without QDs or SPIONs), QDs (without BSA), and SPIONs (without BSA) were measured to be −6 mV, −5 mV, and 20 mV, respectively. The measurements were conducted in the presence of THF with the same mixing ratio as that used in the assembly formation experiments. The negative surface charge of QDs was probably due to the phosphine oxide of the surface ligand TOPO, and the positive surface charge of SPIONs was probably due to the amine group of the surface ligand oleylamine. Thus, the negative surface charge of the supraparticle co-assemblies was probably due to BSA or/and the QDs' surface ligand. The nanoparticles in a supraparticle assembly did not show significant order in spatial distribution (Fig. 1a). In QDs@BSA, packing multiple QDs in a supraparticle co-assembly could result in enhanced fluorescence intensity compared with that of individual QDs (Fig. 1d). It should be noted that the enhancement ratio in fluorescence intensity (Fig. 1d) often appeared to be lower than the encapsulation number of QDs in an assembly (Fig. 1a). This could be due to fluorescence quenching considering that the QDs were densely packed in the assembly as indicated in the TEM images (Fig. 1a). The thermal gravimetric analysis (TGA) results indicated that in a supraparticle co-assembly SPIONs@BSA, the average weight percentages of BSA and SPIONs were 27.1% and 72.9%, respectively (Fig. 1e, ESI note 1†). In addition, it was found that ∼5% of the BSA molecules and ∼90% of the SPIONs added to the initial mixture were incorporated into the supraparticle co-assembly product.

**Fig. 2:**
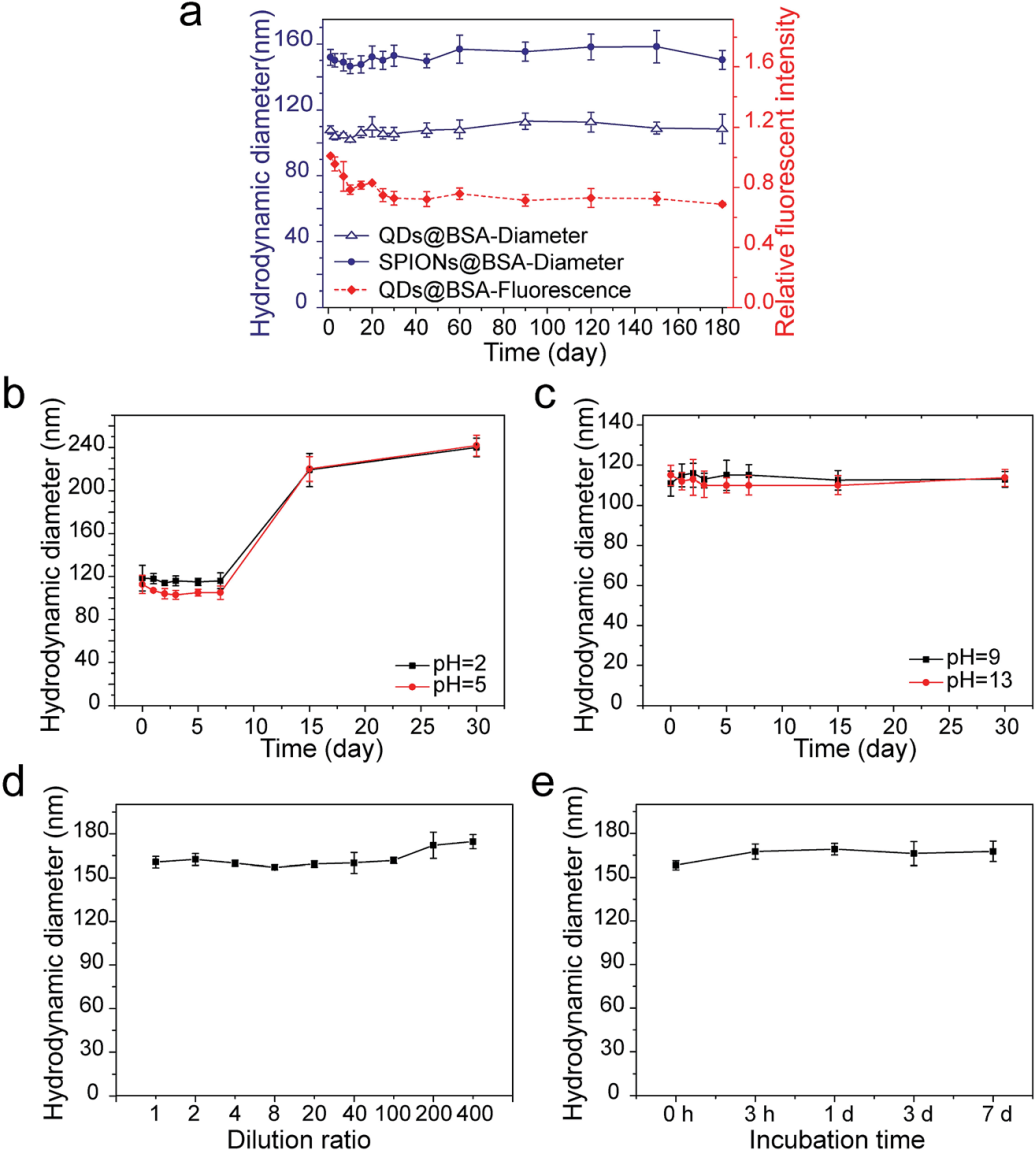
Colloidal stability of supraparticle co-assemblies. (a) Hydrodynamic diameter and fluorescence intensity of supraparticle co-assemblies at 4 °C in PBS (pH 7.4). (b) Hydrodynamic diameter of SPIONs@BSA in acidic environments at 4 °C. (c) Hydrodynamic diameter of SPIONs@BSA in basic environments at 4 °C. (d) Hydrodynamic diameter changes of SPIONs@BSA in response to dilution. Along the *x*-coordinate the dilution ratio increases from the left to the right. The concentration corresponding to dilution ratio 1 is 200 μg mL^−1^. (e) Hydrodynamic diameter of SPIONs@BSA in bovine serum at 37 °C.

The formed supraparticle co-assemblies exhibited outstanding colloidal stability. In PBS at 4 °C, the hydrodynamic sizes of SPIONs@BSA and QDs@BSA have remained virtually unchanged for 6 months and counting (Fig. 2a). The fluorescence intensity of QDs is known to be very sensitive to surface defects of nanoparticles. The fluorescence intensity measurement of QDs@BSA showed changes in the first 25 days, with the fluorescence intensity value on the 25th day being ∼75% of the initial value (Fig. 2a). Afterwards, the fluorescence intensity of QDs@BSA remained nearly unchanged for >5 months (Fig. 2a). Furthermore, the supraparticle co-assemblies demonstrated remarkable stability against harsh environments. In acidic environments (pH 2 and pH 5), the hydrodynamic size of SPIONs@BSA was stable for 7 days (Fig. 2b). The measured size started to increase on the 7th day (Fig. 2b). In basic environments (pH 9 and pH 13), the hydrodynamic size of SPIONs@BSA was stable for >30 days (Fig. 2c). Thus, it appears that the supraparticle co-assemblies possess greater colloidal stability in basic environments compared with acidic environments. Control experiments showed that BSA was colloidally stable for >30 days at all the different pH values tested including acidic, neutral, and basic pH conditions (Fig. S2†). Thus, the lower colloidal stability of the supraparticle co-assemblies in acidic environments compared with neural and basic environments was likely caused by weakened intermolecular forces rather than changes in the protein structure under acidic conditions. Furthermore, the supraparticle co-assemblies also showed great stability against dilution, high temperature (37 °C) and serum (Fig. 2d and e).

The high stability of the supraparticle co-assemblies formed here is remarkable. We speculate the following possible causes for the exceptionally high stability. The driving force of assembly provided here by hydrophobic interaction is likely very strong. The dominant force in forming the three-dimensional structure of globular proteins (*e.g.*, BSA) is hydrophobic interaction.^31^ This suggests that, if a method can be devised to fully take advantage of the hydrophobic regions in proteins, the hydrophobic interaction which drives the co-assembly of proteins and other microscopic entities could be made very strong. In our co-assembly process, the hydrophobic surface of the nanoparticles could induce partial unfolding of the proteins, exposing some of the embedded hydrophobic regions.^38,39^ Subsequently, strong hydrophobic interaction would lead to highly stable supraparticle co-assemblies. Finally, because the aqueous environment of nanoparticles usually has many ions, using electrostatic and van der Waals interactions as the primary driving force for assembly in such an environment would suffer from difficulties in controlling and maintaining force balance in assembly. Thus, using hydrophobic interaction as the primary driving force for assembly could prevent this problem, resulting in improved stability and uniformity of assembly structures.

**Fig. 3:**
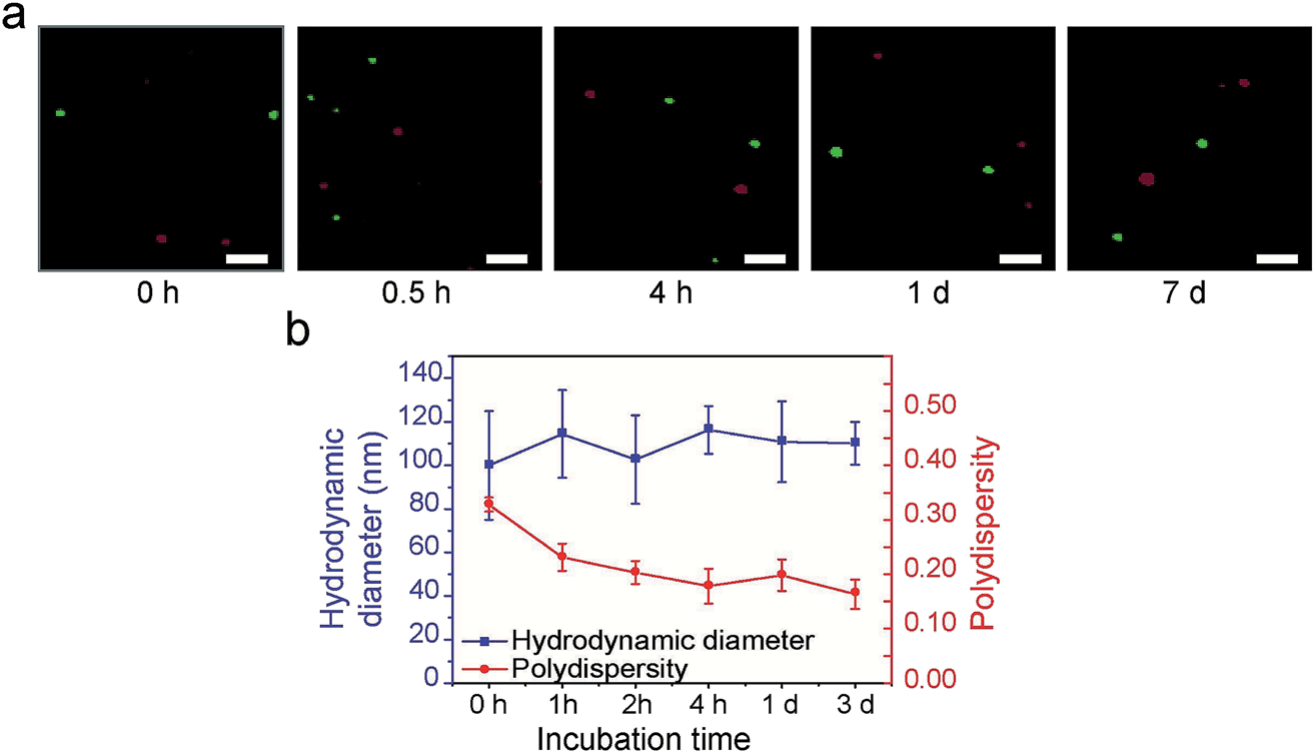
Studies of the formation process of supraparticle co-assemblies. (a) Use of the potential exchange of QDs with different fluorescent colors to examine the formation process of supraparticle co-assemblies. The results indicate that supraparticle co-assemblies form virtually instantly (<1 min; shown as ‘0 h’ in the figure legend). The results also indicate that once the supraparticle co-assemblies are formed, there is no exchange of nanoparticles between different assemblies. Scale bar 5 μm. (b) Use of DLS to examine the formation process of supraparticle co-assemblies. The results indicate that supraparticle co-assemblies form instantly (<1 min; shown as ‘0 h’ in the figure legend). The results also indicate that once the supraparticle co-assemblies are formed, their hydrodynamic diameter does not change significantly (*i.e.*, there is no size growth). The results also indicate that after the supraparticle co-assemblies are formed, there is a gradual decrease in size polydispersity (*i.e.*, there is size focusing).

We performed two lines of experiments to shed more light on the formation process of the supraparticle co-assemblies. First, in two separate containers red and green hydrophobic QDs were mixed with BSA, respectively. Thus, if supraparticle co-assemblies were formed, they were red QDs@BSA and green QDs@BSA, respectively, in the two separate containers. Immediately afterwards (∼1 min), the dispersions in the two separate containers were mixed and incubated, and confocal fluorescence microscopy was performed to examine the mixture (Fig. 3a). It was found that, throughout the incubation process (up to 7 days), the two different colors (red and green) of QDs were never mixed into the same supraparticle co-assembly (Fig. 3a). This finding indicates that supraparticle co-assemblies are formed virtually immediately after mixing hydrophobic nanoparticles and proteins, and that once the supraparticle co-assemblies are formed, the hydrophobic nanoparticles in each supraparticle co-assembly do not exchange with those in other supraparticle co-assemblies in the dispersion.

Second, DLS experiments showed that, immediately (<1 min) after hydrophobic nanoparticles and BSA were mixed, the hydrodynamic diameter value reached that of the supraparticle co-assemblies (∼115 nm), and this value remained largely constant afterwards (Fig. 3b). This result confirms the above finding that the formation of supraparticle co-assemblies is virtually instant. Further, the polydispersity index (PDI) value of the above mixture was found to decrease gradually, starting from ∼0.3 and reaching ∼0.18 in 4 h (Fig. 3b). This result indicates a ‘size focusing’ period after the initial formation of supraparticle co-assemblies. The cause of the ‘size focusing’ is unclear. It is possible that, after the initial instant formation of supraparticle co-assemblies, there is still an exchange of protein molecules between those in the assemblies and the free proteins in PBS.

**Fig. 4:**
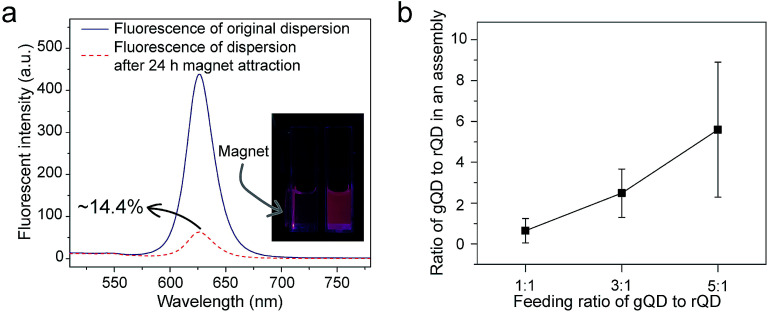
Co-encapsulation of nanoparticles of different types/sizes into supraparticle co-assemblies. (a) Different types of nanoparticles (SPIONs and QDs here) can be co-encapsulated to achieve multifunctionality (superparamagnetism and fluorescence here). (b) The number ratio of different colors (sizes) of QDs co-encapsulated into a supraparticle co-assembly can be controlled by the feed ratio of the assembly ‘reaction’. gQD: green QD; rQD: red QD.

From both fundamental and application points of view, a key question is whether nanoparticles of different types or different sizes can be incorporated into a supraparticle co-assembly together. We mixed hydrophobic SPIONs (dissolved in THF), hydrophobic QDs (dissolved in THF), and BSA (dissolved in PBS). The surface ligand on hydrophobic SPIONs was oleylamine and that on hydrophobic QDs was TOPO. Thus, the chemical components facing outward on the surfaces of hydrophobic SPIONs and hydrophobic QDs were essentially alkyl chains, which were compatible with the hydrophobic domains of proteins composed of nonpolar amino acid side chains. It was found that a large majority (∼85.6%) of the formed supraparticle co-assemblies showed both the fluorescence emitted by QDs and the magnetism exhibited by SPIONs (Fig. 4a). In a transparent container, under the illumination of UV light from a handheld UV lamp, the dispersion of the assemblies exhibited strong fluorescence emitted by QDs, and ∼85.6% of the fluorescent assemblies could be attracted and moved by a permanent magnet, demonstrating the magnetism exhibited by SPIONs (Fig. 4a). This result thus confirms co-encapsulation of SPIONs and QDs in a predominant majority of the supraparticle co-assemblies. In addition, the QD and SPION co-encapsulated supraparticle co-assemblies also showed great colloidal stability (Fig. S6†).

Further, we mixed hydrophobic QDs of green and red fluorescent colors (dissolved in THF) with BSA (dissolved in PBS) to form supraparticle co-assemblies. The two different fluorescent colors of QDs were emitted by different particle sizes. The surface ligand on both green and red hydrophobic QDs was TOPO. Thus, the chemical components facing outward on the surface of green and red hydrophobic QDs were essentially alkyl chains, which were compatible with the hydrophobic domains of proteins composed of nonpolar amino acid side chains. Using a fluorescence microscope we measured the number ratio of green QDs to red QDs in each assembly. Each fluorescent spot in a fluorescence microscopy image was considered to correspond to a supraparticle co-assembly. Aggregation of assemblies in the fluorescence microscopy images was believed to be minimal because different dilutions of the dispersion yielded similar results. We found that the number ratios of green QDs to red QDs in each supraparticle co-assembly were close to those added to the mixture for assembly formation, and the green-to-red number ratio in the assembly ‘product’ could be controlled by varying the green-to-red number ratio used in the ‘reactants’ (Fig. 4b). Previously, in other methods for the production of composite nanoparticles co-encapsulating different types/sizes of nanoparticles, a common problem identified from an application point of view is the lack of control of the co-encapsulation ratio of the different nanoparticles.^32–36^ Here, our experimental results show that, by simply mixing hydrophobic nanoparticles and proteins, composite nanoparticles with great co-encapsulation ratio control could be conveniently and rapidly produced.

**Fig. 5:**
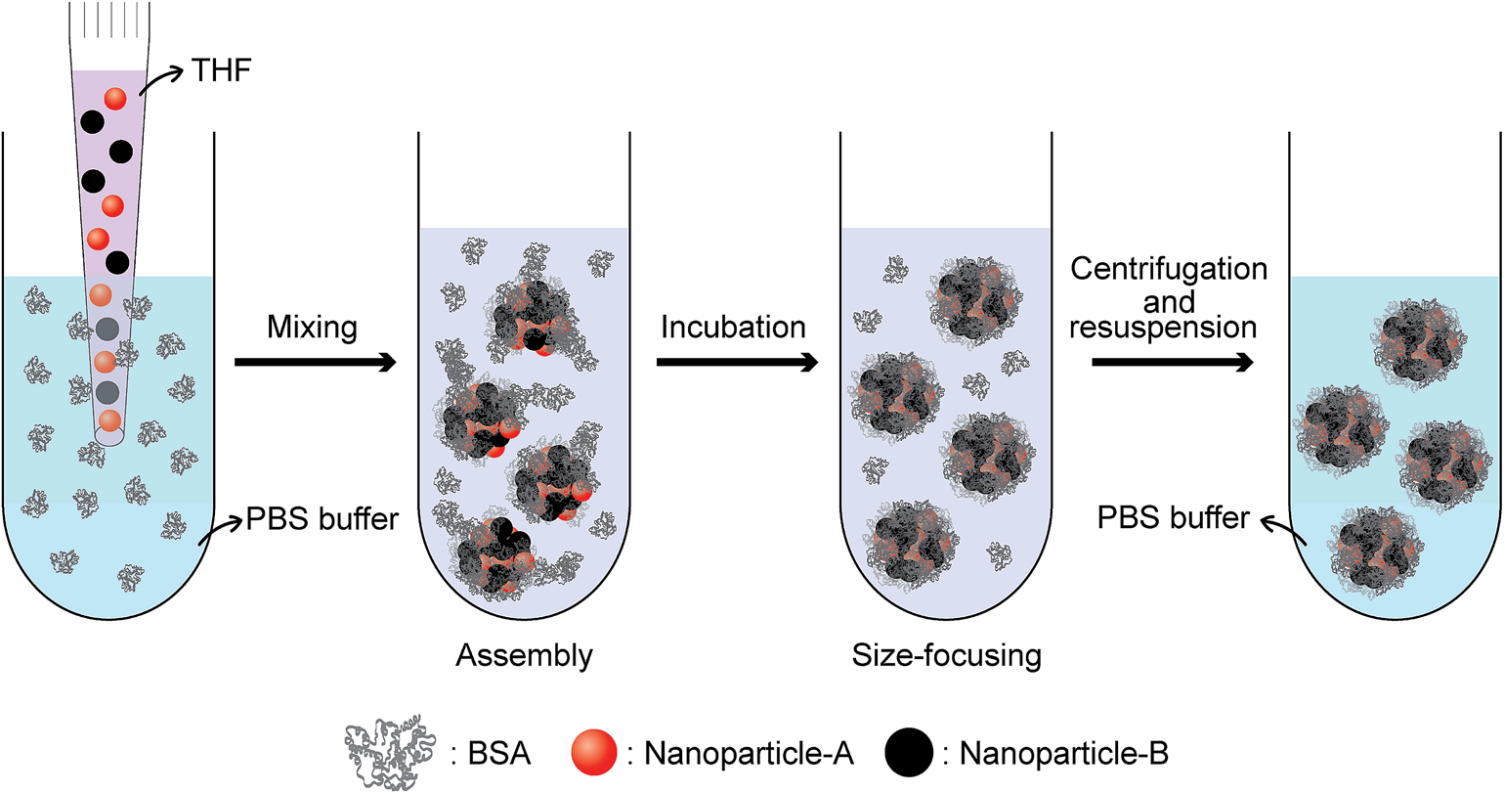
Schematic of the formation process of supraparticle co-assemblies.

Fig. 5 shows a schematic of the formation process of supraparticle co-assemblies suggested by our experimental results. Upon mixing of hydrophobic nanoparticles (dissolved in THF, a water-miscible organic solvent) and the model protein molecule BSA (dissolved in PBS), hydrophobic interaction drives instant formation of highly colloidally stable supraparticle co-assemblies. If different types/sizes of nanoparticles are used, they are co-encapsulated into each assembly with the co-encapsulation ratio well-controlled by the addition ratio. Further incubation of the dispersion results in size focusing (reduction of size polydispersity) of the assemblies. If needed, the assemblies could be separated from free proteins by centrifugation or dialysis and then resuspended. Virtually no BSA molecule is released from the supraparticle co-assemblies as analyzed by UV-Vis light absorption of BSA in solution (Fig. S7†). In addition to BSA (molecular weight 66 430 Da), we also examined another protein bovine α-lactalbumin (BLA, molecular weight 14 178 Da) for the ability to form supraparticle co-assemblies. After mixing with hydrophobic nanoparticles, BLA also showed the capability to rapidly form stable supraparticle co-assemblies (Fig. S8†). This result indicates the generality of the presented assembly process.

It should be mentioned that previously Xie *et al.* used ultraviolet (UV) light to disrupt the disulfide bonds in proteins, exposing the interior hydrophobic regions and thereby triggering the assembly of protein molecules and hydrophobic nanoparticles due to hydrophobic interaction.^40,41^ In contrast, here we show that the presence of hydrophobic nanoparticles alone is sufficient to trigger the hydrophobic interaction-driven assembly of proteins without the need to disrupt any covalent bonds in proteins. In addition, in another separate line of research using hydrophobic interaction to drive protein assembly, some hydrophobic small molecules such as paclitaxel were found to trigger the assembly of proteins.^42,43^ But it was unknown how replacing hydrophobic molecules with hydrophobic nanoparticles would affect the formation, structure, and stability of the assembly. Here, we show that hydrophobic nanoparticles induce rapid formation of highly stable supraparticle co-assemblies, each of which encapsulates multiple nanoparticles and offers good encapsulation ratio control if different nanoparticles are used.

**Fig. 6:**
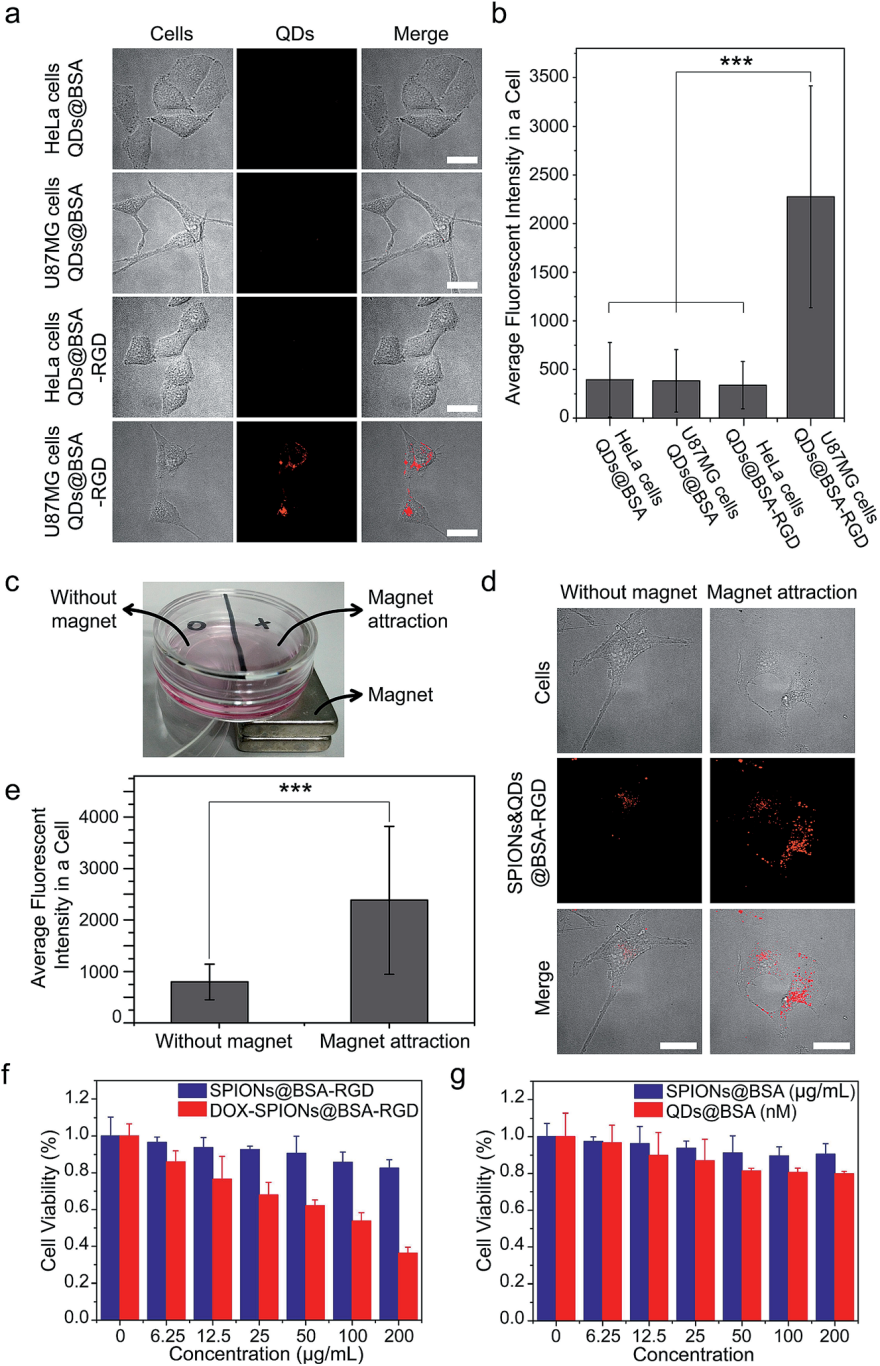
Proof-of-concept studies of biological applications of supraparticle co-assemblies. (a and b) QDs@BSA were conjugated with the RGD peptide, and the formed QDs@BSA-RGD were able to specifically label and image U87MG cells. (a) Shows representative images. (b) Shows quantification results using the images obtained from the experiments of (a). Images of ∼100 cells from each sample were used for the quantification. ***P < 0.001. (c–e) The bi-functionality of SPIONs&QDs@BSA-RGD could be used to achieve combined magnetic targeting and fluorescence imaging of biological cells. (c) Shows the experimental setup. U87MG cells were cultured on the bottom of the dish. SPIONs&QDsBSA-RGD dispersed in the cell culture medium were incubated with the cells. (d) Shows representative images. (e) Shows quantification results using the images obtained from the experiments of (d). Images of ∼100 cells from each sample were used for the quantification. ***P < 0.001. (f) An anticancer drug doxorubicin (DOX) was loaded onto supraparticle co-assemblies to achieve cancer cell-killing effects. The DOX-loaded supraparticle co-assemblies were freshly prepared for the cancer cell-killing experiments (storage time < 1 day). (g) Cell viability studies show that supraparticle co-assemblies are biocompatible.

Finally, we performed several proof-of-concept experiments of biological applications of the supraparticle co-assemblies. First, QDs@BSA were conjugated with the RGD peptide using well-established EDC chemistry.^44^ The formed bioconjugates (QDs@BSA-RGD) could specifically label and image cells with the targeted cell surface receptors (U87MG cells). As shown in Fig. 6a and b, the cellular uptake (indicated by QD fluorescence) of QDs@BSA-RGD into U87MG cells after 12 h was drastically higher than that of the three control samples, namely QDs@BSA into HeLa cells, QDs@BSA into U87MG cells, and QDs@BSA-RGD into HeLa cells after the same duration. This result is consistent with a number of literature reports on the difference in RGD peptide labeling effects between U87MG and HeLa cells.^45–49^ This experiment demonstrates the abilities of the supraparticle co-assemblies for bioconjugation, biological labeling, and biological imaging. Second, hydrophobic SPIONs and QDs were mixed with BSA to form SPION and QD co-encapsulated assemblies, *i.e.*, SPIONs&QDs@BSA. After conjugating the assemblies with the RGD peptide, forming SPIONs&QDs@BSA-RGD, the bioconjugated assemblies were incubated with U87MG cells, and the effect of an external permanent magnet on the cellular uptake of the bioconjugated assemblies was studied. It was found that using an external permanent magnet to attract SPIONs&QDs@BSA-RGD towards the direction of the cells could greatly enhance the cellular uptake of the assemblies after 24 h of incubation of the assemblies with the cells (Fig. 6c–e). This experiment demonstrates the ability of the supraparticle co-assemblies for bi-functional applications, particularly combined magnetic targeting and fluorescence imaging. Third, an anticancer drug doxorubicin (DOX) was loaded onto SPIONs@BSA-RGD by electrostatic interaction. The formed nanostructure, namely DOX-SPIONs@BSA-RGD, was found to kill cancer cells (U87MG cells) in a DOX concentration dependent manner (Fig. 6f). In contrast, without DOX, SPIONs@BSA-RGD did not exhibit significant cancer cell-killing ability under the same experimental conditions (Fig. 6f). It is worth mentioning that the DOX molecules were stable in the supraparticle co-assemblies in PBS at 37 °C for at least 3 days, as measured by the DOX fluorescence (Fig. S9†). Since the DOX-loaded supraparticle co-assemblies were freshly prepared for the cancer cell-killing experiments (storage time < 1 day), the DOX molecules were not released until they reached the interior of cells, where the acidic and enzyme degradative environment in intracellular lysosomes could release DOX from the supraparticle co-assemblies. In addition, the incubation of SPIONs@BSA or QDs@BSA with the cells did not lead to a significant effect on cell viability (Fig. 6g). The cell viability experiments show that supraparticle co-assemblies are biocompatible, and that drug molecules can be incorporated into the supraparticle co-assemblies to provide chemotherapeutic functions.

## Conclusions

In conclusion, we have demonstrated a hydrophobic interaction-driven process to form an emerging class of nanostructures, namely protein–nanoparticle supraparticle co-assemblies. This process can instantly form highly stable assemblies. Different sizes or types of nanoparticles can be co-encapsulated into each supraparticle co-assembly, with the co-encapsulation ratio well-controlled by varying the reactant addition ratio. We speculate that both thermodynamics and kinetics could play a role in determining the structure of the final product. The involvement of thermodynamics could resemble the process reported by the Halas group to form protein–nanoparticle supraparticle co-assemblies using electrostatic interaction, which is driven by balancing of intermolecular forces.^13^ On the other hand, the involvement of kinetics could resemble the nanoprecipitation process.^50,51^ The observations that the injection needle size and flow rate affect the size of the supraparticle co-assemblies are indications that kinetics has a significant involvement in determining the structure of the product. The supraparticle co-assemblies show good biocompatibility and abilities for bioconjugation and multifunctionality. These results suggest the significant potential of applying the supraparticle co-assemblies in biological imaging, delivery, and modulation. Furthermore, these results suggest the significant potential of using hydrophobic interaction to co-assemble proteins with materials, promising very rapid formation of highly stable structures with minimal energy input, all of which are the goals of ideal nano-manufacturing.

## Conflicts of interest

The authors declare no conflict of interest.

## Supplementary Material

NA-001-C9NA00328B-s001
