# Supplementary material for: Spontaneous and instant formation of highly stable protein–nanoparticle supraparticle co-assemblies driven by hydrophobic interaction
Source: Nanoscale Adv. 2019 Sep 26;1(10):4137–47. doi: 10.1039/c9na00328b (PMC9417729; doi:10.1039/c9na00328b)
Supplement: NA-001-C9NA00328B-s001 [file NA-001-C9NA00328B-s001.pdf]

Electronic Supplementary Material (ESI) for Nanoscale Advances.
This journal is © The Royal Society of Chemistry 2019

Supporting information for

Spontaneous and Instant Formation of Highly Stable Protein-Nanoparticle Supraparticle Co-assemblies Driven by Hydrophobic Interaction

Xiaoya Yu [1, 2, 3, 4], *Xiao Liu* [1, 2, 3, 4], *Wanchuan Ding* [1, 2, 3, 4], *Jun Wang* [1, 2, 3, 4], *Gang Ruan* [1, 2, 3, 4, *]

[1] Department of Biomedical Engineering, College of Engineering and Applied Sciences, Nanjing University, China

[2] Institute of Materials Engineering, College of Engineering and Applied Sciences, Nanjing University, China

[3] Collaborative Innovation Center of Chemistry for Life Sciences, Nanjing University, China

[4] Jiangsu Key Laboratory of Artificial Functional Materials, Nanjing University, China

*Correspondence to: gangruan@nju.edu.cn.

Supplementary Notes

Supplementary Note 1: Calculation of the weight percentages of BSA and SPIONs in SPIONs@BSA supraparticle assemblies based on the TGA result.

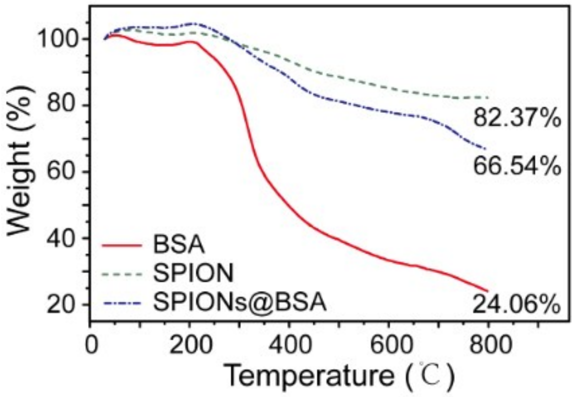

The TGA result is shown above (also in Figure 1e). Suppose the weight percentage of BSA in supraparticle assemblies is x, the weight percentage of SPIONs will be (1-x). Thus, we have the following equation:

$$0.2406x + 0.8237(1 - x) = 0.6654$$

$$x = \frac{0.8237 - 0.6654}{0.8237 - 0.2406} \times 100\%$$

$$x = 27.148\%$$

Thus, the weight percentage of BSA in SPIONs@BSA is determined to be 27.148%, and the weight percentage of SPIONs in SPIONs@BSA is 1-27.148% = 72.852%.

Supplementary Figures

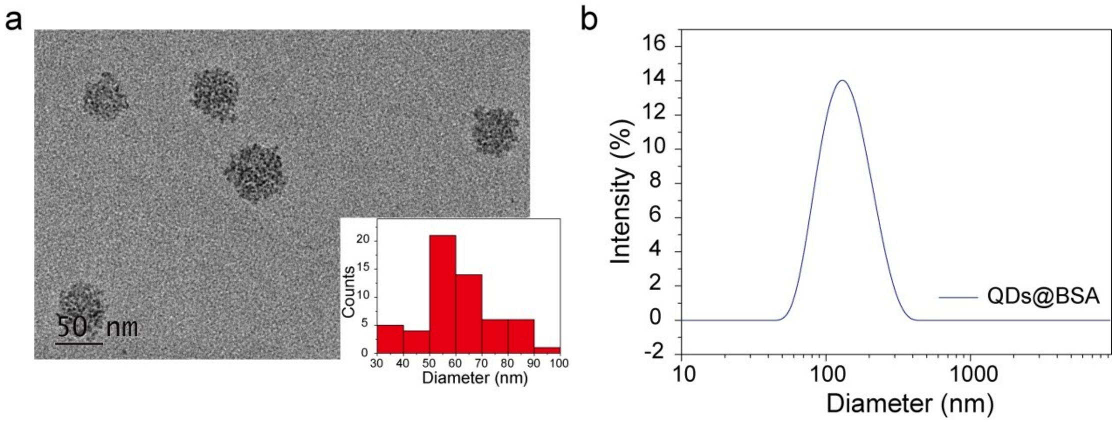

Figure S1 TEM (a) and DLS (b) results of supraparticle co-assemblies formed by mixing hydrophobic QDs (dissolved in THF) and BSA (dissolved in PBS). The formed assemblies are called QDs@BSA here.

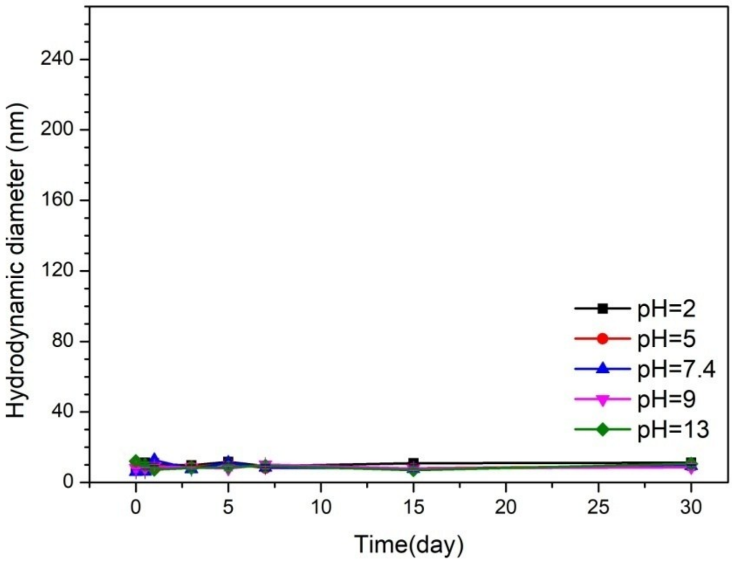

Figure S2 Colloidal stability of BSA in aqueous solution at different pH values. The solution had a small percentage of THF at the same mixing ratio as that used in the assembly formation experiments. It can be seen that BSA is colloidally stable with hydrodynamic diameter ~9 nm for at least 30 days at all the pH values tested, including acidic, neutral, and basic pH values.

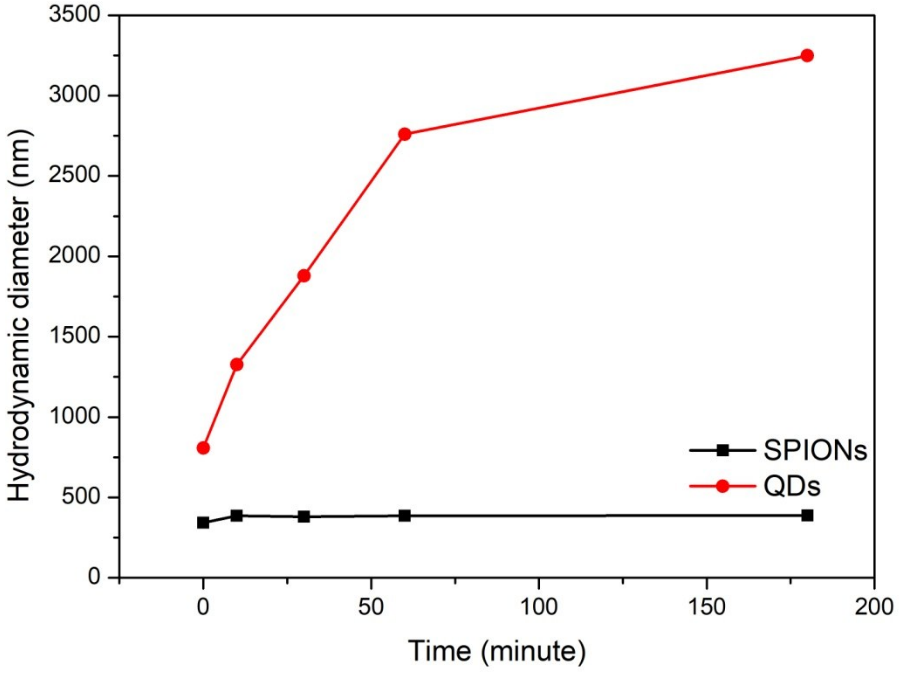

Figure S3 Hydrodynamic size of hydrophobic nanoparticles (SPIONs or QDs) in aqueous environment without proteins. The mixing experiments were conducted the same way as those for supraparticle assembly formation except that no proteins were used. Under the corresponding experimental conditions, presence of BSA would yield co-assemblies with hydrodynamic diameter ~110 nm. In contrast, without BSA, here the data show that the measured hydrodynamic diameter instantly increased to >400 nm.

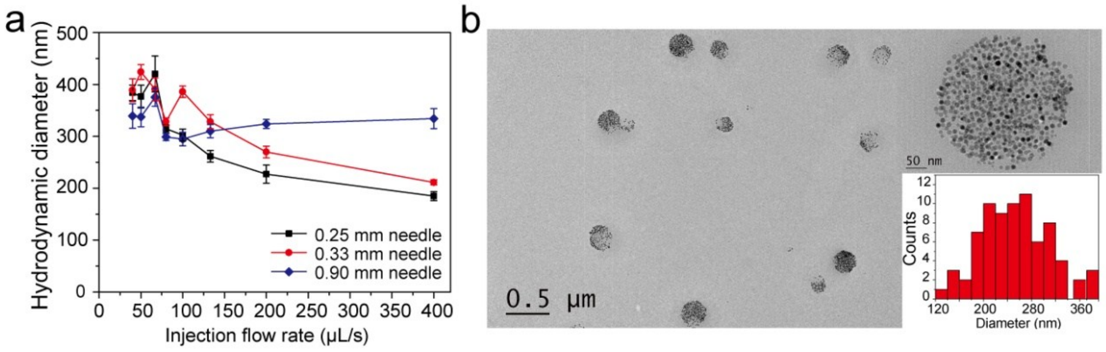

Figure S4 (a) Effects of injection flow rate and injection needle size on the size of the formed supraparticle co-assemblies. Control of injection flow rate was conducted by using a syringe pump. In each experiment 400 μL SPIONs were injected into 3 mL BSA. (b) A TEM image is shown here for assembly sizes much larger than those in the TEM image in Figure 1a of the main text. To obtain larger assembly sizes such as what are shown here, injection rate, injection needle size, or nanoparticle concentration could be adjusted.

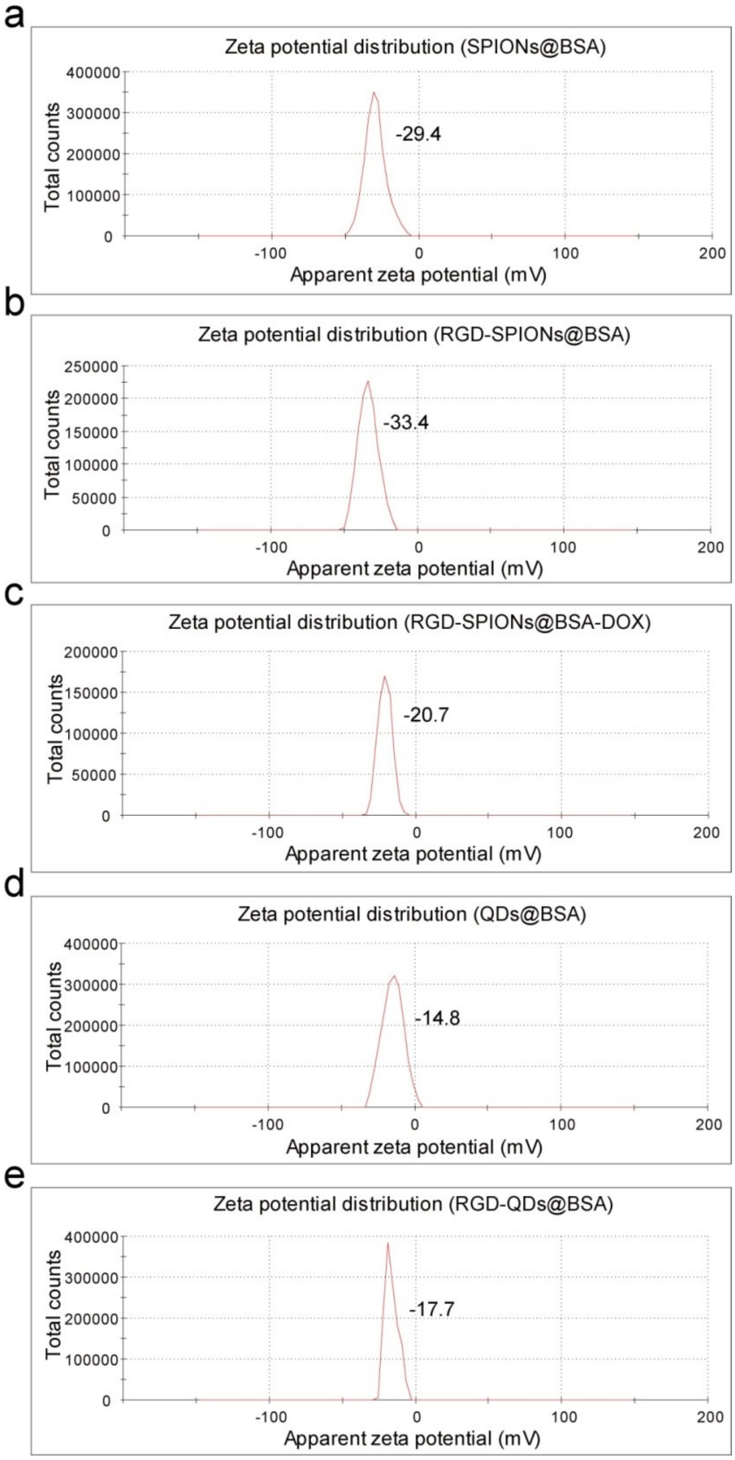

Figure S5 Surface charge (zeta potential) analysis of supraparticle co-assemblies.

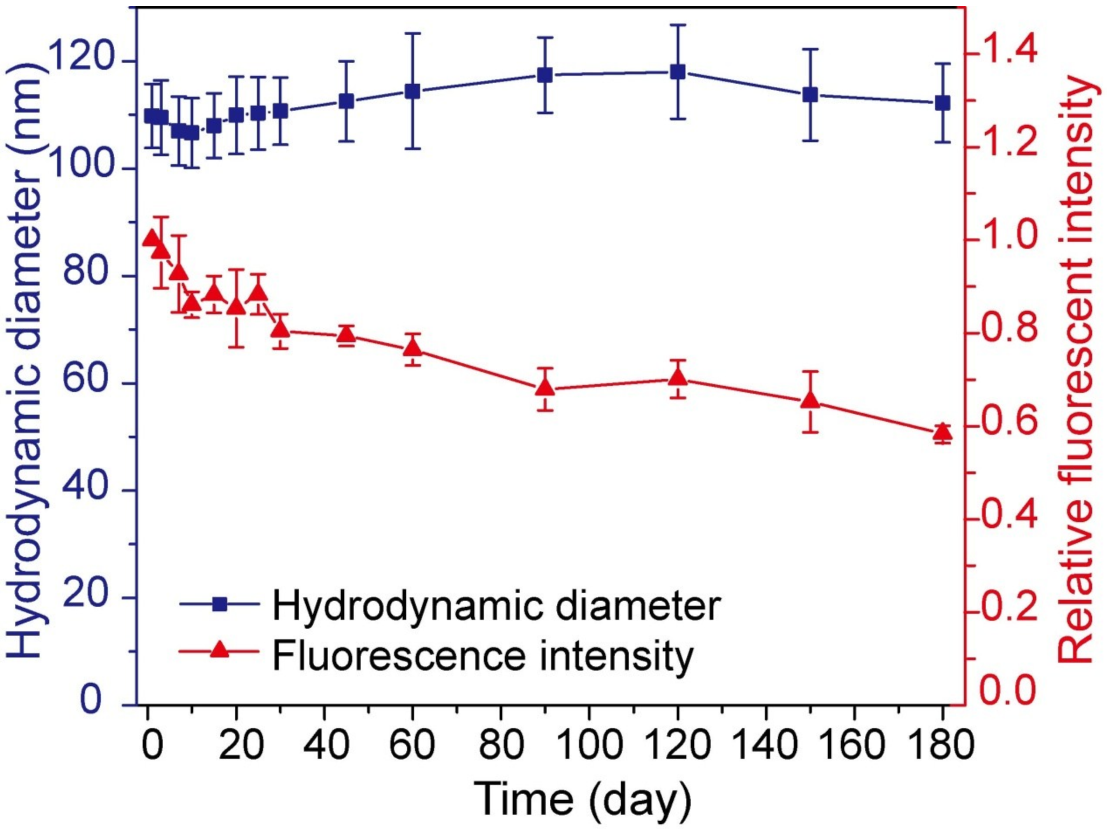

Figure S6 Stability of SPIONs&QDs@BSA (supraparticle co-assemblies with both SPIONs and QDs co-encapsulated).

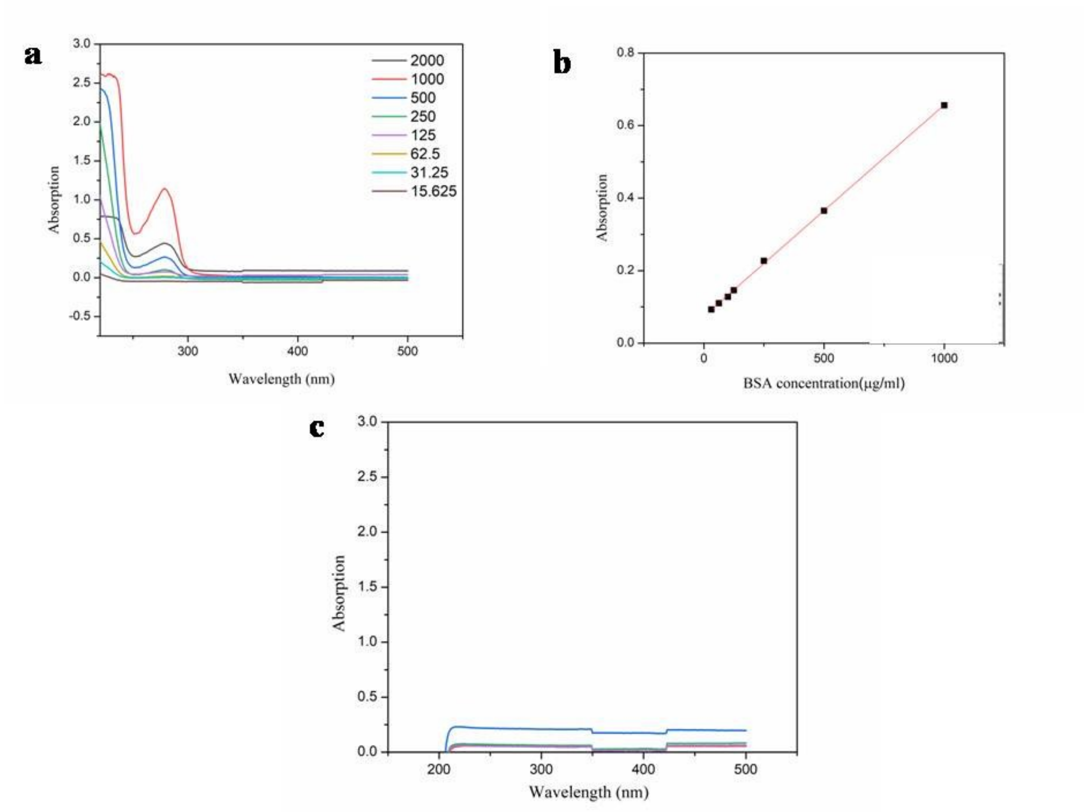

Figure S7 Virtually no BSA molecule is released from supraparticle co-assemblies in solution. SPIONs@BSA were purified by centrifugation and re-suspended in PBS. After one week storage at 4 ºC, the supraparticle co-assemblies were removed by centrifugation, and the supernatant was withdrawn for UV-Vis light absorption measurement for BSA concentration. (a) shows the UV-Vis light absorption spectra of the BSA solutions used for standard curve preparation; (b) shows the standard curve; (c) shows the UV-Vis light absorption spectrum of the supernatant after one week storage of supraparticle co-assemblies, indicating that the BSA amount in the supernatant was below the detection limit, which is ~5% of the BSA in the supraparticle co-assemblies. The three different spectrum curves are from measurements of three replicate supraparticle co-assembly samples.

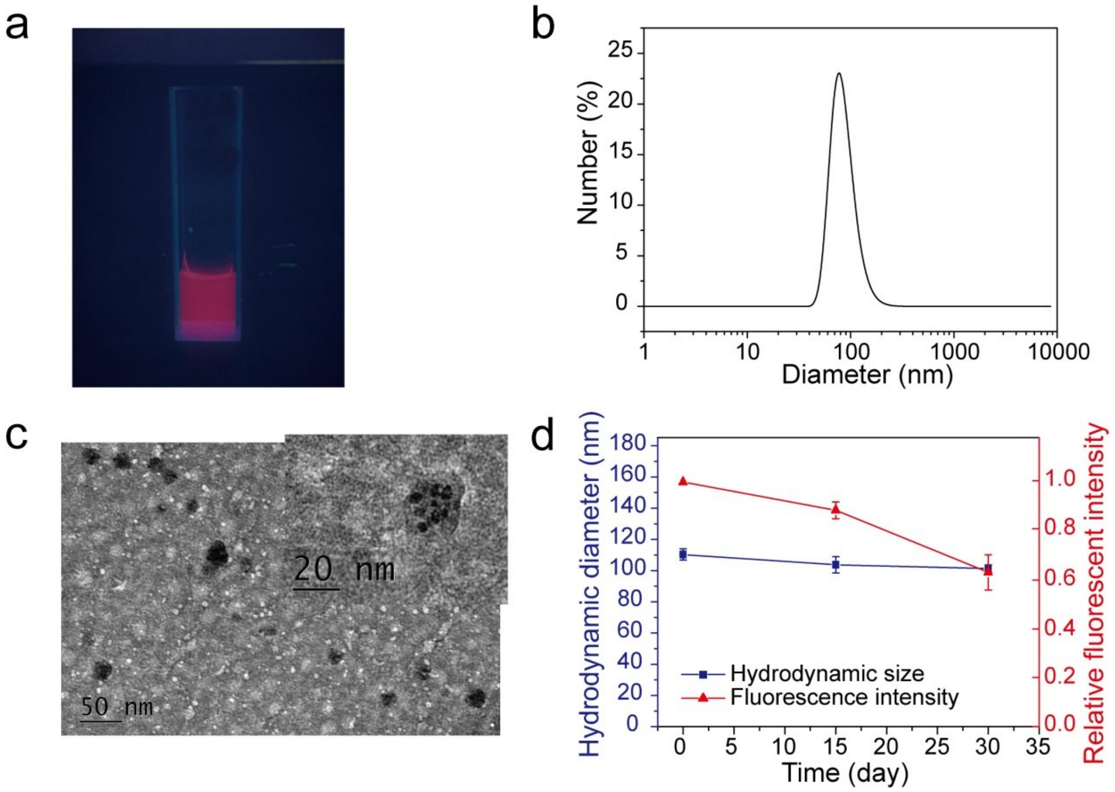

Figure S8 Using bovine α-lactalbumin (BLA) to form supraparticle co-assemblies by mixing BLA with hydrophobic nanoparticles. (a) Fluorescence image of dispersion of QDs@BLA in PBS. (b) Hydrodynamic diameter distribution of QDs@BLA. (c) TEM image. (d) Stability of QDs@BLA dispersed in PBS at 4 °C.

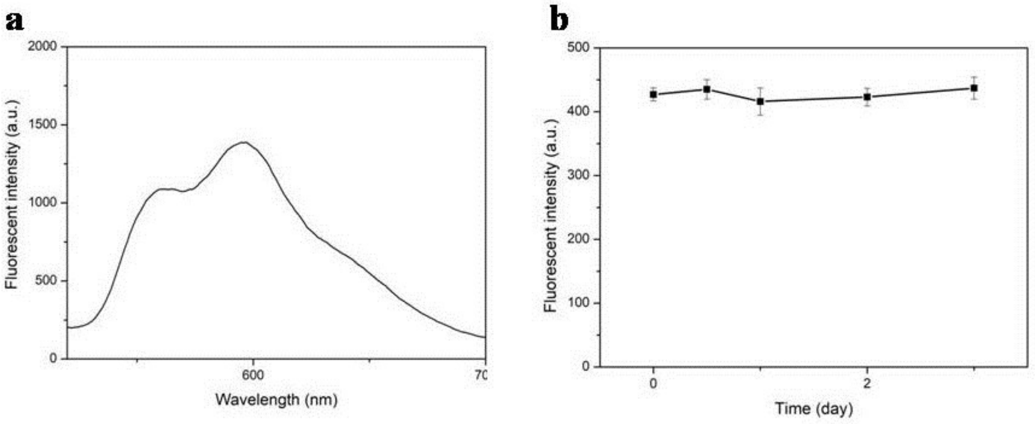

Figure S9 Doxorubicin (DOX) was stable on the DOX-loaded supraparticle co-assemblies. DOX-SPIONs@BSA-RGD supraparticle co-assemblies were placed in PBS at 37 ºC and the change in fluorescent intensity from the DOX on supraparticle co-assemblies was measured over time. At each given time point, the supraparticle co-assemblies were purified by centrifugation and re-suspended in PBS for fluorescence measurement. (a) Fluorescent spectrum of DOX. (b) The change in fluorescent intensity over time.
